# Hypocalcemia in Dialysis Is Not Associated with Increased Mortality: Evidence from a Population-Based Cohort

**DOI:** 10.3390/nu18091386

**Published:** 2026-04-28

**Authors:** Seok Hui Kang, So-Young Park, Yu-Jeong Lim, Bo-Yeon Kim, Ji-Young Choi, Jun-Young Do, Jung-Eun Lee

**Affiliations:** 1 Division of Nephrology, Department of Internal Medicine, College of Medicine, Yeungnam University, Daegu 42415, Republic of Korea; kangkang@ynu.ac.kr; 2 Department of Physiology, College of Medicine, Yeungnam University, Daegu 42415, Republic of Korea; 3 Healthcare Review and Assessment Committee, Health Insurance Review and Assessment Service, Wonju 26465, Republic of Korea; 4 Department of Medicine, Samsung Medical Centre, Sungkyunkwan University School of Medicine, Seoul 06531, Republic of Korea

**Keywords:** calcium, hemodialysis, cardiovascular diseases, hypocalcemia, mortality

## Abstract

**Introduction:** Recent research underscores the risks of maintaining a positive calcium balance in hemodialysis (HD) patients. This study aims to evaluate outcomes based on the calcium levels of HD patients, specifically those with hypocalcemia. **Methods:** In this retrospective cohort study, data from 71,101 HD patients were analyzed and classified into six groups based on calcium levels: severe hypocalcemia (<7.5 mg/dL, *n* = 1078), moderate hypocalcemia (7.5–7.99 mg/dL, *n* = 4000), mild hypocalcemia (8.0–8.39 mg/dL, *n* = 9846), lower-normal calcium (8.4–9.29 mg/dL, *n* = 38,697), upper-normal calcium (9.3–10.19 mg/dL, *n* = 14,505), and hypercalcemia (≥10.2 mg/dL, *n* = 1975). **Results:** The numbers of deaths, CVE, and fracture at the end point of the follow-up were recorded: 401 (37.2%), 189 (23.2%), and 224 (20.8%) in the severe hypocalcemia group, respectively; 1523 (38.1%), 663 (22.8%), and 802 (20.1%) in the moderate hypocalcemia group, respectively; 3985 (40.5%), 1618 (22.9%), and 2054 (20.9%) in the mild hypocalcemia group, respectively; 17,067 (44.1%), 6948 (24.9%), and 8676 (22.4%) in the lower-normal calcium group, respectively; 6904 (47.6%), 2967 (27.3%), and 3471 (23.9%) in the upper-normal calcium group, respectively; and 1074 (54.4%), 457 (30.0%), and 473 (23.9%) in the hypercalcemia group, respectively. The 5-year patient survival rates for the severe hypocalcemia, moderate hypocalcemia, mild hypocalcemia, lower-normal calcium, upper-normal calcium, and hypercalcemia groups were 73.9%, 70.0%, 68.8%, 66.4%, 66.1%, and 62.8%, respectively. The 5-year cardiovascular event-free survival rates for the severe hypocalcemia, moderate hypocalcemia, mild hypocalcemia, lower-normal calcium, upper-normal calcium, and hypercalcemia groups were 78.2%, 79.0%, 78.2%, 76.2%, 75.3%, and 72.6%, respectively. The hazard ratios (HRs) for the all-cause mortality (HR: 0.94, 95% CI: 0.84–1.05) and cardiovascular events (HR: 0.98, 95% CI: 0.84–1.15) of the severe hypocalcemia group were consistently not higher than those of the lower-normal calcium group even after thorough adjustments were made for various clinical variables. Multivariable Cox regression analyses revealed that the HRs for all-cause mortality and cardiovascular events of the mild hypocalcemia groups were lower than those of the lower-normal calcium group. Serum calcium levels were not associated with increased risk of fracture. **Conclusions:** Patients with various degrees of hypocalcemia, including severe hypocalcemia, were not associated with increased mortality and cardiovascular event rates. We suggest that symptoms and clinical presentation should be prioritized rather than simply targeting the normalization of calcium levels in hypocalcemia correction.

## 1. Introduction

Hemodialysis (HD) is the most widely used dialysis modality; globally, the number of patients undergoing HD is rapidly increasing [1]. Efforts have been devoted to reducing the high mortality rate among these patients. Mineral and bone disorder (MBD) is a prevalent and crucial complication in patients on dialysis, profoundly affecting their long-term prognosis. This multifaceted condition involves disruptions in calcium, phosphorus, parathyroid hormone (PTH), and vitamin D metabolism, results in complications such as osteoporosis, bone fractures, vascular calcification, and cardiovascular disease [2,3]. Therefore, the calcium, phosphorus, PTH, and alkaline phosphatase levels of patients on dialysis should be regularly monitored. Healthcare providers should use a comprehensive approach to MBD management that incorporates dietary adjustments, phosphate binders (PPBs), vitamin D supplements, and PTH-regulating medications.

Hypocalcemia often occurs with the progression of chronic kidney disease partly because of the reduced gastrointestinal absorption of calcium due to vitamin D deficiency [4]. Consequently, secondary hyperparathyroidism and MBD occur, historically prompting recommendations to maintain serum calcium within the normal range and correct hypocalcemia. However, studies have highlighted the risks related to maintaining a positive calcium balance in dialysis patients [5,6,7]. These risks include accelerated vascular calcification, increased arterial stiffness, hypertension, and adverse hemodynamic effects. Excess calcium loading may promote calcium deposition in extra-osseous tissues, such as vessels; consequently, it increases the risk of cardiovascular diseases and mortality. This evolving understanding is reflected in the absence of specific treatment directives for hypocalcemia in the 2017 Kidney Disease Improving Global Outcomes Guideline [8].

This study aimed to evaluate clinical outcomes based on various calcium levels in patients undergoing dialysis by using population-based data, specifically hypocalcemia. The findings could help to determine the acceptable range of hypocalcemia and arbitrary target serum calcium levels in patients on dialysis.

## 2. Materials and Methods

### 2.1. Study Population and Database

This retrospective cohort study included adult patients (≥18 years) receiving maintenance HD for at least 3 months with a minimum frequency of two sessions per week. Data were obtained from the fourth through seventh national HD quality assessment programs conducted by the Health Insurance Review and Assessment Service (HIRA) in South Korea, covering the following periods: July and December 2013, July and December 2015, March and August 2018, and October 2020 and March 2021 [9].

Across all assessment cycles, a total of 127,302 individuals were initially identified. To establish a well-defined cohort, patients appearing in more than one assessment cycle were excluded to avoid duplication (*n* = 53,728). Additional exclusions included individuals with unclear dialysis duration or follow-up duration (*n* = 271), those undergoing HD via a catheter (*n* = 1763), patients lacking serum calcium or albumin measurements (*n* = 9), and those with extreme outlier values (1% range on both extremes, *n* = 1430). After applying these criteria, 71,101 patients were eligible for analysis.

The same individual may appear in more than one assessment cycle because the HD quality assessment program includes patients receiving maintenance HD at each HD center during a given 6-month assessment period. To maximize follow-up duration while maintaining patient-level independence, we used data from the earliest assessment cycle as the baseline and did not use data collected in subsequent cycles for the analysis when a patient appeared in two or more assessment cycles. A total of 53,728 patients appeared in more than one assessment cycle. These patients were not excluded from the study population itself; instead, only their repeated assessment data after the first cycle were excluded in the analysis. Although restricting the analysis to the first assessment cycle may introduce some degree of selection bias, our study was not designed to evaluate longitudinal changes in clinical variables over time. Therefore, we considered using a single baseline assessment per patient to be the most appropriate strategy to avoid duplication and within-person correlation. Patients undergoing HD via a catheter were excluded from the analysis. Compared with patients using an arteriovenous fistula or graft, those receiving HD through a catheter more likely represent temporary vascular access situations, such as the interruption or failure of a pre-existing access or other clinical conditions that are not fully captured in the database. Because catheter use may reflect acute illness, unstable clinical status, or unmeasured complications, unknown confounding effects could be introduced by the inclusion of these patients, and the study results could be potentially influenced. Therefore, we excluded catheter-based HD patients to maintain a more homogeneous study population.

The study included patients who participated in the 4th, 5th, 6th, and 7th HD quality assessment programs. These programs generally targeted prevalent HD patients receiving maintenance HD at least twice weekly for at least 3 months in HD centers. Therefore, our study population included maintenance HD patients, and we did not specifically exclude the patients undergoing long-term dialysis. The mean dialysis duration was 51.2 ± 57.7 months, with an interquartile range of 48 months.

We calculated serum calcium levels by using the corrected calcium level with total calcium and serum albumin. We excluded the patients with at least one extreme outlier value (1%) for serum calcium. The 1% threshold was selected to balance two competing considerations: minimizing the influence of extreme values while retaining sufficient sample size and representativeness. Given the large sample size of our nationwide cohort, excluding the uppermost and lowermost 1% was considered a pragmatic and conservative approach commonly used in epidemiologic studies to reduce the influence of skewed distributions and potential recording errors without materially altering the underlying population structure. We also excluded the patients with unclear dialysis duration or follow-up duration (*n* = 271). Specifically, we excluded the patients for whom the end point of follow-up could not be identified from the claim data (*n* = 2), patients with a calculated follow-up duration of ≤0 days based on the claim data (*n* = 41), and patients whose HD duration recorded in the assessment program was ≤3 months (*n* = 228).

This study was approved by the Institutional Review Board of Yeungnam University Medical Center (YUMC 2023-12-012, 15 December 2023) and conducted in accordance with the Declaration of Helsinki. The requirement for informed consent was waived because all data were anonymized prior to analysis.

### 2.2. Study Variables

Information on demographic and clinical characteristics, including sex, body mass index (BMI), age, dialysis duration (months), the presence of diabetes as the underlying cause of end-stage kidney disease, and type of vascular access, was collected. Clinical parameters such as ultrafiltration volume (L per session), hemoglobin (g/dL), BMI (kg/m^2^), Kt/V_urea_, serum albumin (g/dL), phosphorus (mg/dL), and creatinine (mg/dL) were obtained as part of the routine assessment. These variables were measured on a monthly basis, and mean values were calculated using all available measurements during the assessment period. Kt/V_urea_ was estimated using the Daugirdas formula [10]. Each of the 4th–7th HD quality assessment programs covered an identical 6-month assessment period. Laboratory parameters were recorded monthly, and the mean value of all available measurements during the assessment period was used as the baseline value for each patient. When one or more monthly measurements were missing among the six expected values, the baseline value was calculated as the mean of the remaining available measurements. The baseline variables of Kt/V_urea_ (*n* = 1769), ultrafiltration volume (*n* = 14), and hemoglobin (*n* = 1) were completely missing; specifically, 1784 values were missing across these variables. For patients with unavailable value for a given variable during the assessment period, median imputation was used for that specific variable. Because the proportion of completely missing data was smaller than the total study population, we believe that this approach unlikely affected the study findings.

Serum calcium levels were defined as the corrected calcium level by using the following equation: corrected calcium level (mg/dL) = total calcium (mg/dL) − 0.8 × [serum albumin (g/dL) − 4.0] [11]. The patients were divided into six groups based on the serum calcium levels: the severe hypocalcemia group with <7.5 mg/dL; the moderate hypocalcemia group with 7.5–7.99 mg/dL; the mild hypocalcemia group with 8.0–8.39 mg/dL; the lower-normal calcium group with 8.4–9.29 mg/dL, the upper-normal calcium group with 9.3–10.19 mg/dL; and the hypercalcemia group with ≥10.2 mg/dL. Hypocalcemia was categorized into three groups based on its severity guided by serum calcium levels to determine the acceptable levels of hypocalcemia in dialysis patients.

Medication codes are provided in Table S1. We assessed the use of several drug classes: renin–angiotensin system blockers (RASB), antiplatelet agents (clopidogrel and aspirin), cinacalcet, statins, and vitamin D analogs such as paricalcitol. In addition, calcium-containing supplements (e.g., calcium gluconate, citrate, lactate, and phosphate) and PPB were evaluated, which were further categorized into calcium-based (calcium carbonate or calcium acetate) and non-calcium-based agents (lanthanum or sevelamer). Medication exposure was defined as a prescription duration of at least 30 days. Comorbid conditions were quantified using the Charlson Comorbidity index (CCI), calculated based on ICD-10 codes recorded during the year preceding the HD quality assessment. CCI scores were derived according to established methods [12,13,14]. The CCI score was calculated on the basis of the presence of the predefined 16 comorbid conditions identified using ICD-10 codes as described in a previous study [14]. The following points were given: 1 point for myocardial infarction (MI), congestive heart failure (CHF), peripheral vascular disease, cerebrovascular disease, dementia, chronic pulmonary disease, rheumatologic disease, peptic ulcer disease, mild liver disease, and diabetes without complications; 2 points for diabetes with complications, hemiplegia or paraplegia, and any malignancy; 3 points for moderate to severe liver disease; and 6 points for metastatic tumor and acquired immune deficiency syndrome/human immunodeficiency virus. The total CCI score was calculated as the sum of these weighted comorbid conditions; higher scores indicated a greater comorbidity burden.

### 2.3. Outcomes

Participants were followed through June 2024. The primary endpoint was all-cause mortality, while cardiovascular events (CVEs) and fractures were evaluated as secondary outcomes. Mortality data were obtained from the HIRA database. Patients who switched to peritoneal dialysis or underwent transplantation before experiencing an event were censored at the time of modality change.

CVEs, including MI, stroke, and revascularization procedures, were identified irrespective of survival status, following previously established definitions [9,14]. To assess incident events, patients with a prior diagnosis of each condition within one year before the HD quality assessment were excluded. Fracture outcomes were defined using ICD-10 diagnostic codes recorded during follow-up, including femur (S72), wrist and hand (S62), forearm (S52), lumbar spine and pelvis (S32), and rib, sternum, and thoracic spine (S22) [15].

### 2.4. Statistical Analyses

Statistical analyses were performed using SAS Enterprise Guide (version 7.1; SAS Institute, Cary, NC, USA) and R software (version 3.5.1; R Foundation for Statistical Computing, Vienna, Austria). Categorical variables are presented as counts with percentages, and continuous variables are expressed as mean ± standard deviation. Group differences for categorical variables were assessed using the chi-square test or Fisher’s exact test, as appropriate. Continuous variables across groups were compared using one-way ANOVA with Tukey’s post hoc test for multiple comparisons.

Survival probabilities were estimated using the Kaplan–Meier method and compared using the log-rank test. Hazard ratios (HRs) with corresponding 95% confidence intervals (CIs) were calculated using Cox proportional hazards models. Multivariable models were adjusted for potential confounders, including BMI, CCI score, sex, age, vascular access type, dialysis duration, diabetes status, ultrafiltration volume, Kt/V_urea_, hemoglobin, albumin, creatinine, phosphorus levels, presence of MI or CHF, and use of relevant medications (RASBs, cinacalcet, vitamin D agents, calcium supplements, aspirin, clopidogrel, PPBs, and statins). We plotted the spline curves of the possibility of the non-linear association between calcium levels and clinical outcomes. We used the same covariates in spline models to explore the nonlinear associations between calcium levels and outcomes. All variables were entered simultaneously into the model. Statistical significance was set at *p* < 0.05.

## 3. Results

### 3.1. Clinical Characteristics

The baseline characteristics are shown in Table 1.

The patients in the severe hypocalcemia group were the youngest and had the highest ultrafiltration volume, serum phosphorus, and serum creatinine levels. The proportion of males decreased as the calcium level increased. The upper-normal calcium and hypercalcemia groups had lower proportions of diabetes and usage of calcium supplements and RASBs but had higher proportions of usage of cinacalcet or vitamin D agents than the other groups. These two groups also had higher HD vintages, Kt/V_urea_, and hemoglobin levels but had lower body mass index and serum albumin level than the other groups. The hypercalcemia group had the lowest proportions of MI or CHF and usage of statins or calcium PPB but had lower CCI scores than the other groups.

### 3.2. Effect of Serum Calcium Levels on Deaths, CVE, and Fracture

The follow-up durations for the severe hypocalcemia, moderate hypocalcemia, mild hypocalcemia, lower-normal calcium, upper-normal calcium, and hypercalcemia groups were 66 ± 36, 63 ± 34, 61 ± 34, 62 ± 35, 66 ± 38, and 67 ± 40 months, respectively. The numbers of deaths, CVE, and fracture at the end point of the follow-up were recorded: 401 (37.2%), 189 (23.2%), and 224 (20.8%) in the severe hypocalcemia group, respectively; 1523 (38.1%), 663 (22.8%), and 802 (20.1%) in the moderate hypocalcemia group, respectively; 3985 (40.5%), 1618 (22.9%), and 2054 (20.9%) in the mild hypocalcemia group, respectively; 17,067 (44.1%), 6948 (24.9%), and 8676 (22.4%) in the lower-normal calcium group, respectively; 6904 (47.6%), 2967 (27.3%), and 3471 (23.9%) in the upper-normal calcium group, respectively; and 1074 (54.4%), 457 (30.0%), and 473 (23.9%) in the hypercalcemia group, respectively.

**Figure 1:**
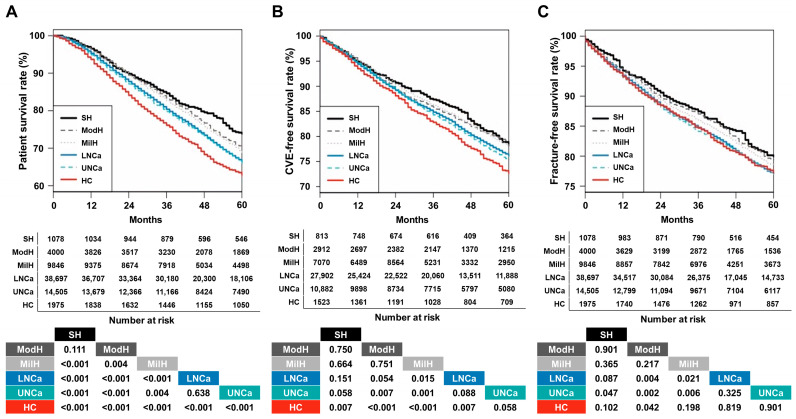
Kaplan–Meier curves for patient survival, cardiovascular events, or fracture according to groups. (**A**) Patient survival. (**B**) Cardiovascular events. (**C**) Fracture. *p*-values for pairwise comparison with log-rank tests were added to the bottom of the graph. Abbreviations: CVE, cardiovascular events; SH, severe hypocalcemia; ModH, moderate hypocalcemia; MilH, mild hypocalcemia; LNCa, lower-normal calcium; UNCa, upper-normal calcium; HC, hypercalcemia.

The proportions of death at the end point of the follow-up increased as the serum calcium level increased. The 5-year patient survival rates of the severe hypocalcemia, moderate hypocalcemia, mild hypocalcemia, lower-normal calcium, upper-normal calcium, and hypercalcemia groups were 73.9%, 70.0%, 68.8%, 66.4%, 66.1%, and 62.8%, respectively (Figure 1A).

The 5-year CVE-free survival rates for the severe hypocalcemia, moderate hypocalcemia, mild hypocalcemia, lower-normal calcium, upper-normal calcium, and hypercalcemia groups were 78.2%, 79.0%, 78.2%, 76.2%, 75.3%, and 72.6%, respectively (Figure 1B). The 5-year fracture-free survival rates for the severe hypocalcemia, moderate hypocalcemia, mild hypocalcemia, lower-normal calcium, upper-normal calcium, and hypercalcemia groups were 80.0%, 79.4%, 78.5%, 77.1%, 77.0%, and 77.2%, respectively (Figure 1C). None of the three hypocalcemia groups showed unfavorable survival curves for the three outcomes compared with the patients with normal calcium levels.

Table 2 displays the unadjusted or adjusted HRs for deaths, CVE, and fractures in each of the groups compared with those in the lower-normal calcium group.

The HRs for all-cause mortality (HR: 0.94, 95% CI: 0.84–1.05, *p* = 0.242) and CVE (HR: 0.98, 95% CI: 0.84–1.15, *p* = 0.865) for the severe hypocalcemia group were not higher than those for the lower-normal calcium group even after thorough adjustments were made for various clinical variables. Multivariable Cox regression analyses revealed that the HRs for all-cause mortality and CVE for the mild hypocalcemia group were lower than those for the lower-normal calcium group. Serum calcium levels were not associated with increased risk of fracture.

In the spline curves using a univariable model, patients with low calcium levels had a trend of having a lower risk on all-cause mortality, CVEs, and fracture; furthermore, those with high calcium levels exhibited a trend of having a higher risk on these outcomes than the median values (Figure S1). In the spline curves using a multivariable model, these trends were maintained in all-cause mortality and CVE.

### 3.3. Effect of Serum Calcium Levels on Deaths, CVE, and Fracture: Subgroup Analyses

**Figure 2:**
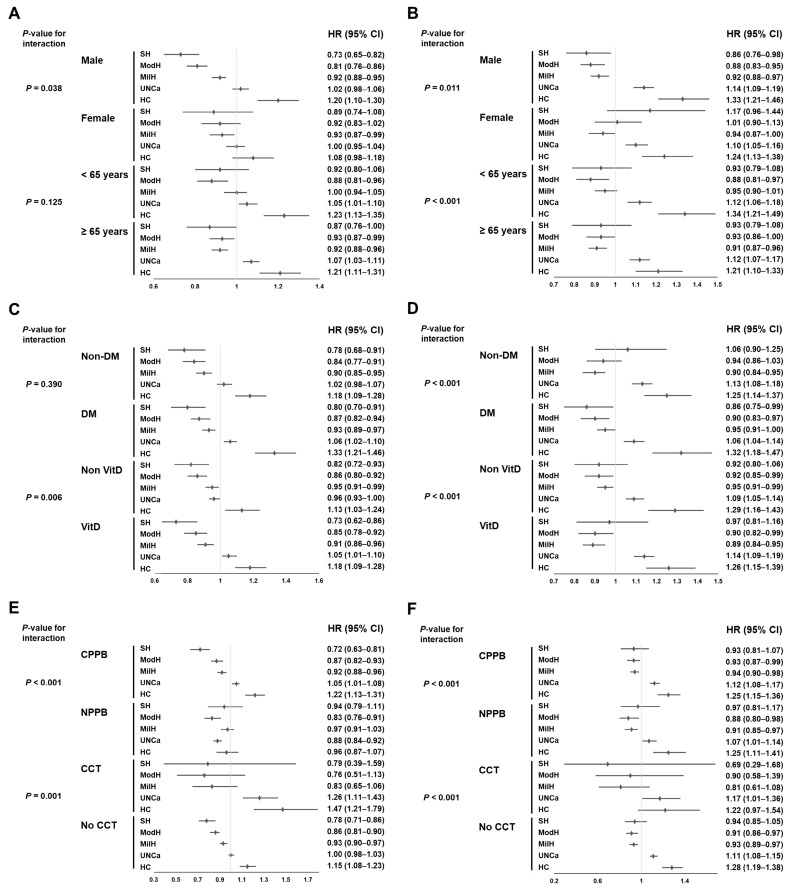
Forest plots of hazard ratios and 95% confidence intervals of all-cause mortality according to subgroups. Univariable (**A**, **C**, or **E**) and multivariable (**B**, **D**, or **F**) analyses. The LNCa group served as the reference group. Adjustments were made according to the following factors: age; sex; body mass index; DM; the Charlson comorbidity index score; vascular access type; hemodialysis vintage; ultrafiltration volume; Kt/V_urea_; hemoglobin, albumin, creatinine, and phosphorus levels; the administration of renin–angiotensin system blockers, CCT, VitD, calcium supplement, aspirin, clopidogrel, phosphate binder, and statins; and myocardial infarction or congestive heart failure. Abbreviations: CVE, cardiovascular events; HR, hazard ratio; SH, severe hypocalcemia; ModH, moderate hypocalcemia; MilH, mild hypocalcemia; LNCa, lower-normal calcium; UNCa, upper-normal calcium; HC, hypercalcemia; VitD, vitamin D analog; DM, diabetes mellitus; CPPB, calcium-based phosphate binder; NCPPB, non-calcium-based phosphate binder; CCT, cinacalcet.

Subgroup analyses were performed on the basis of sex, age, presence of diabetes, or use of vitamin D analogs, type of PPBs, or cinacalcet. In multivariable analyses, the risk of all-cause mortality in the severe hypocalcemia group did not increase compared with that in the lower-normal calcium group in all subgroups (Figure 2).

The all-cause mortalities for the mild hypocalcemia and moderate hypocalcemia groups were lower than those for the lower-normal calcium group in most subgroups although the difference was not significant in some subgroups. The risk of all-cause mortality increased in the upper-normal calcium and hypercalcemia groups compared with that in the lower-normal calcium group across all subgroups, and a marginal difference was observed in patients who used cinacalcet.

The protective effects on CVE risk for the severe hypocalcemia, moderate hypocalcemia, and mild hypocalcemia groups compared with that for the lower-normal calcium group lost significance in several subgroups, but no evidence of increased risk was found (Table S2). Conversely, the increased risk in the upper-normal calcium and hypercalcemia groups compared with that in the lower-normal calcium group was notably significant in most subgroups, particularly those without calcium PPB or without cinacalcet compared with the groups that received these medications. However, serum calcium levels did not consistently affect the fracture risk in most subgroups.

### 3.4. Sensitivity Analyses

We divided the patients into quartiles. The calcium levels were 8.10 ± 0.31 mg/dL in the first quartile (1Q), 8.67 ± 0.11 mg/dL in the second quartile (2Q), 9.04 ± 0.12 mg/dL in the third quartile (3Q), and 9.70 ± 0.35 mg/dL in the fourth quartile (4Q). Figure S2 shows patient survival, CVE-free, and fracture-free rates according to the quartiles of calcium level. The 5-year patient survival rates for the 1Q, 2Q, 3Q, and 4Q groups were 68.9%, 66.7%, 66.1%, and 65.8%, respectively. The 5-year CVE-free survival rates for the 1Q, 2Q, 3Q, and 4Q groups were 78.0%, 76.0%, 76.5%, and 74.9%, respectively. The 5-year fracture-free survival rates for 1Q, 2Q, 3Q, and 4Q groups were 78.7%, 77.5%, 76.6%, and 76.9%, respectively. Kaplan–Meier curves show the best patient, CVE-free, and fracture-free rates in 1Q among the quartiles. Cox regression analyses revealed similar trends for three outcomes (Table S3).

## 4. Discussion

This study was conducted with a large sample of HD patients. The results showed that individuals with hypocalcemia exhibited overall favorable outcomes in all-cause mortality and CVE risk compared with patients who had corrected calcium levels of 8.4–9.3 mg/dL. Even in cases of severe hypocalcemia (<7.5 mg/dL), the all-cause mortality and CVE risk did not increase compared with those in cases with serum calcium levels of 8.4–9.3 mg/dL. Additionally, the mortality and CVE risk of individuals with mild to moderate hypocalcemia (7.5–8.4 mg/dL) were lower than those of individuals with calcium levels of 8.4–9.3 mg/dL regardless of the use of vitamin D analogs or calcium-containing PPBs. Furthermore, we found no association between hypocalcemia and fracture risk.

This study primarily aimed to evaluate the lower limit of serum calcium levels that are acceptable without adverse long-term effects on patients undergoing dialysis. However, achieving this objective was challenging because increased mortality rates were not observed in patients with various degrees of hypocalcemia, including those with severe hypocalcemia (defined as <7.5 mg/dL, 1.5% of total subjects). Nevertheless, our findings suggested that hypocalcemia should be corrected in terms of symptoms and clinical presentation rather than solely aiming to normalize the calcium levels. Notably, the severe hypocalcemia group had a higher proportion of younger patients who could tolerate hypocalcemia or remain asymptomatic, and could therefore maintain lower calcium levels. Additionally, the mortality and CVE risk of individuals with mild to moderate hypocalcemia were lower than those of the individuals with calcium levels of 8.4–9.3 mg/dL, and they did not show an increased risk of fracture. These findings suggested that the criteria for hypercalcemia which required a reduction in the dosage of vitamin D analogs or calcium-containing PPBs, may be reduced [16].

The association between hypocalcemia and the risk of mortality in HD patients remains controversial. However, more studies have indicated that hypocalcemia is associated with poor prognosis. Several studies using data from Davita and DOPPS have reported such an association [17,18]. These findings might not be completely aligned with our results possibly because of the difference in data sources. Previous studies utilized data from patients undergoing HD in the early 2000s, while our study focused on data from patients receiving HD between 2013 and 2021. During this period, the use of calcimimetics and lower calcium concentrations in dialysate became more widespread. Therefore, our results might be more relevant to current HD patients.

The evidence explaining the association between hypocalcemia and favorable outcomes is inconclusive. However, among patients undergoing dialysis, those who experience less frequent positive calcium balance that results in sustained hypocalcemia may have better prognoses. The positive calcium balance in dialysis patients primarily arises from a higher dialysate calcium concentration than the patient’s serum calcium levels and from the use of calcium-based PPBs and vitamin D derivatives. This positive calcium balance can cause adverse outcomes such as vascular calcification and arterial stiffening, adynamic bone disease, soft tissue calcification, and potentially higher mortality risk, particularly in patients already at a high cardiovascular risk or with existing calcifications. Most of the clinical manifestations associated with a positive calcium balance can also be observed in patients with high Ca × P levels or uncontrolled secondary hyperparathyroidism. Therefore, the favorable outcomes observed in patients with hypocalcemia in our study might reflect relatively lower Ca × P burden or less advanced secondary hyperparathyroidism rather than the calcium level itself. Patients with chronic hypocalcemia tend to maintain increased PTH levels, which promote bone resorption and help to preserve near-normal serum calcium concentrations. Uncontrolled secondary hyperparathyroidism is more commonly associated with hypercalcemia and an increased fracture risk because of excessive bone resorption. This interpretation might be supported by the more favorable outcomes observed particularly in younger patients, as well as the lack of association between hypocalcemia and fracture risk in our study. To provide more objective evidence, researchers should perform further analysis incorporating Ca × P values and PTH levels to help to determine whether the prognostic differences according to calcium levels are indirect findings reflecting differences in Ca × P burden and secondary hyperparathyroidism severity or whether calcium itself directly affects outcomes.

Individuals with serum calcium levels between 9.3 and 10.2 mg/dL exhibited increased risks of all-cause mortality and CVE compared with those of individuals with lower serum calcium levels, although such values are within the normal range. Moreover, individuals with hypercalcemia (≥10.2 mg/dL) exhibited even higher risks. In HD patients, hypercalcemia influences the occurrence of cardiovascular disease and cardiovascular death through mechanisms such as endothelial dysfunction and vascular or valvular calcification [19,20]. A recent study has also demonstrated that hypercalcemia can influence infection-related deaths through changes in host defense mechanisms [21]. Consistent with previous findings, our results showed similar trends. For CVE, serum calcium levels of ≥9.3 mg/dL were associated with an increased risk in some subgroups. A statistically significant interaction was observed in the subgroups in terms of PPB or cinacalcet. These associations may be explained by two factors. First, treatment-associated hypercalcemia may be ruled out in patients receiving non-calcium-containing PPB or no PPB. In such cases, hypercalcemia may be related to a poorer prognosis. Second, the sample size of patients with cinacalcet was small. A small sample size in the subgroup may be associated with non-significant results compared with the groups without cinacalcet (*n* = 39 in the severe hypocalcemia group; *n* = 144 in the moderate hypocalcemia group; *n* = 388 in the mild hypocalcemia group; *n* = 1814 in the lower-normal calcium group; *n* = 1421 in the upper-normal calcium group; *n* = 301 in the hypercalcemia group).

Normal calcium levels vary depending on the center and measurement method, but they are generally considered to range from approximately 8.4 mg/dL to 10.2 mg/dL [22]. We regarded this range as normal and further divided it into lower-normal calcium (8.4–9.29 mg/dL) and upper-normal calcium (9.3–10.19 mg/dL) according to the median value within the normal range. Symptoms of hypocalcemia generally occur more frequently when serum calcium levels fall below 7.5 mg/dL; therefore, we defined <7.5 mg/dL as severe hypocalcemia. The range between severe hypocalcemia and normal calcium was further divided on the basis of approximate median values into moderate hypocalcemia (7.5–7.99 mg/dL) and mild hypocalcemia (8.0–8.39 mg/dL). Serum calcium levels of ≥10.2 mg/dL were defined as hypercalcemia. Although we established these categories for convenience, we also performed sensitivity analyses by using calcium level as a continuous variable. We analyzed the quartiles of serum calcium level or spline curve.

In our study, medication exposure, defined as “≥30 days of prescription”, did not necessarily indicate continuous use throughout the entire follow-up period. Instead, it was intended to identify patients with meaningful baseline exposure to the medication during the assessment period. Drug exposure was defined as a fixed baseline variable based on prescriptions recorded during the HD quality assessment period and was not modeled as a time-varying exposure during follow-up. Therefore, patients were classified according to whether they had received the medication for at least 30 days during the baseline period, and follow-up period was calculated from the end point of the 6-month HD quality assessment program. We acknowledge that this approach may introduce immortal time bias because patients must survive long enough to accumulate at least 30 days of prescription exposure to be classified as exposed. However, because medication use was defined during the baseline assessment period rather than after cohort entry, we believe that the magnitude of this bias is likely limited. Furthermore, this definition aimed to distinguish chronic users from patients who received only very short-term or incidental prescriptions.

Studies on the adverse effects of calcium-based PPBs and several epidemiologic studies have demonstrated the risks associated with hypercalcemia [17,23,24]. However, studies have yet to clarify whether hypocalcemia is truly harmful and what the optimal calcium target should be in patients undergoing maintenance HD. Epidemiologic studies have suggested that hypocalcemia is generally associated with worse outcomes than the reference calcium range [17,24]. Ter Meulen et al. showed that low-calcium dialysate may also have harmful effects, including QT prolongation, ventricular arrhythmia, intradialytic hypotension, and sudden cardiac death [25]. However, other studies have suggested that non-severe hypocalcemia may have a neutral association with mortality; furthermore, lower calcium exposure through the use of low-calcium dialysate may provide potential clinical benefits by attenuating vascular calcification, arterial stiffness, and endothelial dysfunction [26,27,28]. In patients with low bone turnover or adynamic bone disease, maintaining relatively lower calcium levels may be beneficial because excess calcium exposure may accelerate extraosseous calcification. Allowing relatively lower calcium levels within an acceptable range may be clinically beneficial according to an individual’s chronic kidney disease–MBD status. The serum-to-dialysate calcium gradient may have greater clinical significance than the calcium dialysate concentration [25]. Thus, selecting the calcium dialysate concentration according to the patient’s serum calcium level, rather than applying a uniform dialysate calcium prescription to all patients, may improve clinical outcomes. Therefore, these findings suggest that serum calcium targets should not be determined uniformly. Instead, calcium management should be individualized through a more comprehensive approach that considers multiple factors, including biochemical parameters, intact PTH levels, bone turnover status, and overall chronic kidney disease–MBD status. Given the established risks related to hypercalcemia, maintaining calcium levels within a broader acceptable range that may even include non-severe hypocalcemia, combined with an individualized selection of dialysate calcium concentration, may represent an optimal strategy to improve outcomes in patients undergoing maintenance HD.

## 5. Limitations

Our study had some limitations. First, a retrospective observational design was used in our study. Data were collected for the assessment of HD centers without specific research objectives. However, our dataset might provide a depiction more closely aligned with real-world scenarios, contrasting with data collected for research purposes. Second, our study did not include laboratory findings (such as ionized calcium or intact PTH levels) and clinical findings (such as dialysate calcium level or data on vascular calcification). Serum calcium level was defined as albumin-corrected calcium by using serum calcium and albumin levels measured in each HD center without standardization for laboratory studies. Serum calcium level was also defined as the average levels over a limited period (6 months) during the HD quality assessment program. Third, the diagnosis of CVE or fracture was evaluated using the ICD-10 code or procedures, but data on the cause of death were not included. The diagnosis of events in accordance with the ICD-10 code may differ from actual diagnosis, and the ICD-10 code may be entered without an actual event. In addition, our study presented the death status but not the data on the cause of death. We evaluated all-cause mortality and CVE; however, we were unable to analyze cardiovascular mortality separately because detailed data on the cause of death were unavailable in our dataset. Therefore, deaths were analyzed irrespective of their specific causes. The assessment of cause-specific mortality, particularly cardiovascular mortality, could provide additional insights into the relationship between the calcium status and adverse outcomes in patients undergoing HD. Disturbances in calcium balance may contribute to various pathologies, such as vascular calcification, arrhythmia, and hemodynamic instability, and cardiovascular mortality analysis may help to clarify the potential mechanisms underlying the observed associations. Furthermore, our study involved a retrospective observational design, and causal inferences regarding the relationship between hypocalcemia severity and mortality could not be made. The residual confounding and misclassification of mortality risk may still be present. Future prospective studies, including detailed cause-of-death information, should be performed to better define the association between calcium abnormalities and cardiovascular mortality; these data would help to determine the occurrence of death and allows us to understand the mechanisms by which calcium levels influence adverse outcomes. Fourth, the significant differences in baseline characteristics and sample sizes among the groups could be a cause of selection bias. Although multivariable or subgroup analyses were performed using various factors to minimize this bias, eliminating the effects of all confounding factors was challenging.

## 6. Conclusions

Our study found that patients with various degrees of hypocalcemia, including severe hypocalcemia, were not associated with increased mortality and CVE rates. These results suggested that symptoms and clinical presentation should be prioritized rather than simply targeting the normalization of calcium levels in hypocalcemia correction. Additionally, the mortality and CVE risks of individuals with mild-to-moderate hypocalcemia were lower than those with calcium levels of 8.4–9.3 mg/dL without an increased risk of fractures. These findings also implied that the criteria for hypercalcemia, which may prompt a reduction in vitamin D analog or calcium-containing PPB dosage, might need reconsideration. However, considering the limitations of this study, further research should incorporate factors such as ionized calcium, PTH levels, vascular calcification, or data on dialysate to obtain a clearer conclusion.

## 7. Practical Application

This study’s findings have the following practical applications:

1. Non-severe hypocalcemia in maintenance HD patients was not associated with increased mortality, cardiovascular events, or fracture risk and may not require aggressive correction solely to normalize laboratory values.
2. Clinical symptoms and overall patient status might be prioritized over rigid calcium normalization, particularly in patients with asymptomatic hypocalcemia.
3. Upper-normal calcium and hypercalcemia were related to worse outcomes. This finding is consistent with previous recommendations, suggesting that excessive calcium loading should be used more cautiously.

## Figures and Tables

**Table 1:** Patient clinical characteristics according to serum calcium levels.

	SH Group (*n* = 1078)	ModH Group (*n* = 4000)	MilH Group (*n* = 9846)	LN Group (*n* = 38,697)	UN Group (*n* = 14,505)	HC Group (*n* = 1975)	*p*
**Demographics**							
Age (years)	57.8 ± 13.2	59.8 ± 13.0 ^a^	61.1 ± 13.0 ^ab^	61.6 ± 13.2 ^abc^	60.1 ± 12.9 ^acd^	59.9 ± 12.5 ^acd^	<0.001
Sex (male, %)	804 (74.6%)	2954 (73.9%)	6976 (70.9%)	23,383 (60.4%)	7299 (50.3%)	971 (49.2%)	<0.001
HD vintage (months)	42 ± 49	37 ± 44	37 ± 44	46 ± 53 ^bc^	73 ± 69 ^abcd^	99 ± 79 ^abcde^	<0.001
BMI (kg/m^2^)	23.3 ± 3.8	23.0 ± 3.6	22.8 ± 3.4 ^a^	22.7 ± 3.5 ^abc^	22.4 ± 3.5 ^abcd^	22.0 ± 3.4 ^abcde^	<0.001
**Comorbidities**							
Diabetes mellitus	481 (44.6%)	1890 (47.3%)	4977 (50.5%)	18,343 (47.4%)	5806 (40.0%)	626 (31.7%)	<0.001
CCI score	7.7 ± 3.1	8.0 ± 3.0 ^a^	8.1 ± 3.0 ^a^	7.8 ± 3.0 ^c^	7.2 ± 3.1 ^abcd^	6.7 ± 3.0 ^abcde^	<0.001
**Dialysis-related indices**							
Arteriovenous fistula	947 (87.8%)	3468 (86.7%)	8467 (86.0%)	32,805 (84.8%)	12,308 (84.9%)	1680 (85.1%)	<0.001
Kt/V_urea_	1.45 ± 0.25	1.47 ± 0.25	1.48 ± 0.26 ^ab^	1.53 ± 0.27 ^abc^	1.57 ± 0.28 ^abcd^	1.59 ± 0.28 ^abcd^	<0.001
UFV (L/session)	2.6 ± 1.0	2.5 ± 0.9 ^a^	2.4 ± 0.9 ^ab^	2.3 ± 1.0 ^abc^	2.3 ± 0.9 ^abc^	2.3 ± 0.9 ^ab^	<0.001
**Laboratory findings**							
Hemoglobin (g/dL)	10.6 ± 0.8	10.6 ± 0.8	10.6 ± 0.7	10.7 ± 0.8 ^bc^	10.7 ± 0.8 ^abcd^	10.7 ± 0.9 ^abc^	<0.001
Albumin (g/dL)	4.11 ± 0.41	4.06 ± 0.34 ^a^	4.04 ± 0.32 ^ab^	3.98 ± 0.32 ^abc^	3.95 ± 0.33 ^abcd^	3.92 ± 0.33 ^abcde^	<0.001
P (mg/dL)	5.4 ± 1.5	5.1 ± 1.3 ^a^	4.9 ± 1.2 ^ab^	4.8 ± 1.2 ^abc^	5.0 ± 1.3 ^abcd^	5.2 ± 1.3 ^acde^	<0.001
CR (mg/dL)	10.3 ± 2.9	9.8 ± 2.7 ^a^	9.4 ± 2.7 ^ab^	9.2 ± 2.8 ^abc^	9.7 ± 2.7 ^acd^	9.9 ± 2.6 ^acde^	<0.001
**Medications**							
Use of RASB	693 (64.3%)	2790 (69.8%)	6846 (69.5%)	25,526 (66.0%)	8840 (60.9%)	1151 (58.3%)	<0.001
Use of cinacalcet	39 (3.6%)	144 (3.6%)	388 (3.9%)	1814 (4.7%)	1421 (9.8%)	301 (15.2%)	<0.001
Use of VitD agent	472 (43.8%)	1796 (44.9%)	4448 (45.2%)	18,241 (47.1%)	7680 (52.9%)	1231 (62.3%)	<0.001
Use of CaS	47 (4.4%)	149 (3.7%)	375 (3.8%)	1217 (3.1%)	366 (2.5%)	30 (1.5%)	<0.001
Use of aspirin	262 (24.3%)	1090 (27.3%)	2960 (30.1%)	10,807 (27.9%)	3103 (21.4%)	329 (16.7%)	<0.001
Use of clopidogrel	157 (14.6%)	649 (16.2%)	1788 (18.2%)	6288 (16.2%)	1850 (12.8%)	177 (9.0%)	<0.001
Use of statins	468 (43.4%)	1874 (46.9%)	4927 (50.0%)	18,547 (47.9%)	6000 (41.4%)	669 (33.9%)	<0.001
MI or CHF	541 (50.2%)	2069 (51.7%)	5036 (41.1%)	18,636 (48.2%)	6144 (42.4%)	774 (39.2%)	<0.001
Use of PPB							<0.001
No	163 (15.1%)	614 (15.4%)	1522 (15.5%)	5551 (14.3%)	1717 (11.8%)	260 (13.2%)	
Calcium PPB	716 (66.4%)	2650 (66.3%)	6587 (66.9%)	26,249 (67.8%)	9630 (66.4%)	1171 (59.3%)	
Non-calcium PPB	199 (18.5%)	736 (18.4%)	1737 (17.6%)	6897 (17.8%)	3158 (21.8%)	544 (27.5%)	

Data are expressed as mean ± standard deviation for continuous variables and as numbers (percentages) for categorical variables. *p*–values are tested using one-way analysis of variance, followed by Tukey’s post hoc test, and Pearson’s χ^2^ test for categorical variables. Abbreviations: SH, severe hypocalcemia with serum calcium <7.5 mg/dL; ModH, moderate hypocalcemia with serum calcium of 7.5–7.99 mg/dL; MilH, mild hypocalcemia with serum calcium of 8.0–8.39 mg/dL; LN, lower normal group with serum calcium of, 8.4–9.29 mg/dL; UN, upper-normal calcium with serum calcium of 9.3–10.19 mg/dL; HC, hypercalcemia wirh serum calcium ≥10.2 mg/dL; BMI, body mass index; CaS, calcium supplement; CCI, Charlson comorbidity index; CHF, congestive heart failure; CR, serum creatinine; HD, hemodialysis; Kt/V_urea_, dialysis adequacy calculated using K (dialyzer urea clearance), t (dialysis time), and V (volume of urea distribution); MI, myocardial infarction; P, serum phosphorus; RASB, renin–angiotensin system blockers; PPB, phosphate binder; UFV, ultrafiltration volume; VitD, vitamin D. ^a^ *p* < 0.05 vs. Severe hypocalcemia group, ^b^ *p* <0.05 vs. Moderate hypocalcemia group, ^c^ *p* <0.05 vs. Mild hypocalcemia group, ^d^ *p* <0.05 vs. Lower-normal calcium group, and ^e^ *p* < 0.05 vs. Upper-normal calcium group.

**Table 2:** Cox regression analyses for all-cause mortality, cardiovascular event, or fracture.

	Univariable		Multivariable	
	HR (95% CI)	*p*	HR (95% CI)	*p*
**All-cause mortality**				
Severe hypocalcemia	0.78 (0.71–0.87)	<0.001	0.94 (0.84–1.05)	0.242
Moderate hypocalcemia	0.86 (0.81–0.90)	<0.001	0.91 (0.86–0.96)	0.001
Mild hypocalcemia	0.94 (0.90–0.97)	<0.001	0.93 (0.89–0.96)	<0.001
Lower-normal calcium	reference			
Upper-normal calcium	0.99 (0.97–1.02)	0.598	1.12 (1.08–1.15)	<0.001
Hypercalcemia	1.12 (1.05–1.19)	<0.001	1.28 (1.19–1.37)	<0.001
**Cardiovascular event**				
Severe hypocalcemia	0.89 (0.77–1.03)	0.121	0.98 (0.84–1.15)	0.865
Moderate hypocalcemia	0.92 (0.85–0.99)	0.029	0.93 (0.85–1.02)	0.105
Mild hypocalcemia	0.93 (0.88–0.98)	0.007	0.90 (0.85–0.95)	<0.001
Lower-normal calcium	reference			
Upper-normal calcium	1.04 (0.99–1.09)	0.067	1.10 (1.05–1.15)	<0.001
Hypercalcemia	1.16 (1.05–1.27)	0.003	1.30 (1.17–1.44)	<0.001
**Fracture**				
Severe hypocalcemia	0.87 (0.76–0.99)	0.041	1.04 (0.90–1.20)	0.633
Moderate hypocalcemia	0.88 (0.82–0.95)	<0.001	0.97 (0.89–1.04)	0.382
Mild hypocalcemia	0.93 (0.89–0.98)	0.006	0.97 (0.92–1.02)	0.201
Lower-normal calcium	reference			
Upper-normal calcium	1.02 (0.98–1.06)	0.253	1.02 (0.98–1.07)	0.341
Hypercalcemia	1.02 (0.93–1.12)	0.723	1.00 (0.90–1.11)	0.958

Severe hypocalcemia, <7.5 mg/dL; Moderate hypocalcemia, 7.5–7.99 mg/dL; Mild hypocalcemia, 8.0–8.39 mg/dL; Lower-normal calcium, 8.4–9.29 mg/dL (reference); Upper-normal calcium, 9.3–10.19 mg/dL; Hypercalcemia, ≥10.2 mg/dL. Multivariable analysis was adjusted for age, sex, body mass index, diabetes, vascular access, hemodialysis vintage, Charlson comorbidity index score, ultrafiltration volume, Kt/V_urea_, hemoglobin, serum albumin, serum creatinine, serum phosphorus, use of renin–angiotensin system blockers, cinacalcet, vitamin D analogs, calcium supplement, aspirin, clopidogrel, phosphate binder, and statins, myocardial infraction or congestive heart failure, and was performed using enter mode. The lower-normal calcium group served as the reference. Abbreviations: CI, confidence interval; HR, hazard ratio.

## Data Availability

The raw data were generated at the Health Insurance Review and Assessment Service. The database can be requested from the Health Insurance Review and Assessment Service by sending a study proposal, including the purpose of the study, study design, and duration of analysis, to the website (https://www.hira.or.kr) (accessed on 22 April 2026). The authors cannot distribute the data without permission.

## Supplementary Materials

The following supporting information can be downloaded at https://www.mdpi.com/article/10.3390/nu18091386/s1. Table S1: Medication types and Health Insurance Review and Assessment Service codes; Table S2: Cox regression analyses for cardiovascular event or fracture in subgroups; Table S3: Cox regression analyses for all-cause mortality, cardiovascular event, or fracture based on the quartiles of calcium level; Figure S1: Spline curves show the hazard ratios and 95% confidence intervals of clinical outcomes according to calcium level; Figure S2: Kaplan–Meier curves of patient survival, cardiovascular events, or fracture according to the quartiles of calcium level.
