# Supplementary material for: Hypocalcemia in Dialysis Is Not Associated with Increased Mortality: Evidence from a Population-Based Cohort
Source: Nutrients. 2026 Apr 28;18(9):1386. doi: 10.3390/nu18091386 (PMC13164701; doi:10.3390/nu18091386)
Supplement: Supplementary file 1 [file nutrients-18-01386-s001.zip › nutrients-4239416-supplementary.pdf]

Table S1. Medication types and Health Insurance Review and Assessment Service codes

Table S2. Cox regression analyses for cardiovascular event or fracture in subgroups

Table S3. Cox regression analyses for all-cause mortality, cardiovascular event, or fracture based on the quartiles of calcium level

Figure S1. Spline curves show the hazard ratios and 95% confidence intervals of clinical outcomes according to calcium level

Figure S2. Kaplan–Meier curves of patient survival, cardiovascular events, or fracture according to the quartiles of calcium level

Table S1. Medication types and Health Insurance Review and Assessment Service codes

Medications	Codes
Alacepril	104201ATB, 104202ATB
Benazepril	114701ATB
Captopril	122901ATB, 122902ATB, 122903ATB
Cilazapril	133001ATB, 133002ATB, 133003ATB
Enalapril	151601ATB, 151603ATB
Fosinopril	163501ATB, 163502ATB
Imidapril	173401ATB, 173402ATB
Moexipril	196801ATB, 196802ATB
Lisinopril	184501ATB
Perindopril	211301ATB, 211302ATB, 501601ATB, 501602ATB
Quinapril	221901ATB,
Ramipril	222401ATB, 222402ATB, 222404ATB
Zofenopril	510401ATB, 510402ATB, 510403ATB
Temocapril	235002ATB
Delapril	140901ATB, 140902ATB
Captopril + Hydrochlorothiazide	262200ATB, 262300ATB
Enalapril + Hydrochlorothiazide	440300ATB, 453700ATB, 453600ATB
Ramipril + Felodipine	447100ATB, 447200ATB
Ramipril + Hydrochlorothiazide	448600ATB, 448700ATB
Perindopril + indapamide	556200ATB
Lisinopril + Hydrochlorothiazide	499200ATB, 499300ATB
Moexipril + Hydrochlorothiazide	440800ATB, 497900ATB
Enalapril + nitrendipine	466000ATB
Candesartan	122601ATB, 122602ATB, 122603ATB
Irbesartan	177301ATB, 177303ATB
Losartan	185701ATB, 185702ATB
Valsartan	247101ATB, 247102ATB, 247103ATB, 247104ATB
Fimasartan	515201ATB, 515202ATB, 515203ATB
Azilsartan	662401ATB, 662402ATB, 662403ATB
Telmisartan	378801ATB, 378802ATB
Eprosartan	429201ATB

Olmesartan	468501ATB, 468502ATB, 468503ATB, 520901ATB, 520902ATB
Valsartan + Amlodipine	492800ATB, 492900ATB, 495800ATB, 522600ABTB, 522700ABTB, 522800ABTB, 522900ABTB, 523000ATB, 523100ATB, 523200ATB, 523300ATB, 523400ATB
Valsartan + Lercanidipne	522200ATB. 522300ATB. 522400ATB
Valsartan + Pitavastatin	634900ATB, 635000ATB, 635100ATB, 635200ATB
Valsartan + Sacubitril	651401ATB, 651402ATB, 651403ATB
Valsartan + Rosuvastatin	629700ATB, 629800ATB, 525000ATB, 525100ATB, 525200ATB, 525300ATB,
Valsartan + Hydrochlorothiazide	356400ATB, 442600ATB
Olmesaetan + Amlodipine	500500ATB, 500600ATB, 547500ATB. 547600ATB, 547700ATB, 547800ATB, 547900ATB, 548000ATB, 582200ATB, 582400ATB, 629400ATB, 629500ATB, 629600ATB, 631300ATB, 632800ATB, 632900ATB, 633000ATB
Olmesartan + Hydrochlorothiazide	513600ATB
Olmesartan + Hydrochlorothiazide + Amlodipine	519700ATB, 519800ATB, 519900ATB, 520000ATB, 520100ATB
Olmesartan + Rosuvastatin	653200ATB, 644100ATB, 644200ATB, 526300ATB, 526400ATB, 526500ATB, 526900ATB
Telmisartan + Hydrochlorothiazide	502600ATB, 443200ATB, 443300ATB
Telmisartan + Rosuvastatin	629900ATB, 630000ATB, 630100ATB, 630200ATB, 631600ATB, 631700ATB
Telmisartan + Amlodipine	511500ATB, 511600ATB, 511700ATB, 521200ATB, 521300ATB, 521400ATB, 623100ATB, 644800ATB
Telmisartan+ Hydrochlorothiazide + Amlodipine	663500ATB, 663600ATB, 663700ATB, 663800ATB
Telmisartan + Rosuvastatin + Amlodipine	671700ATB, 671600ATB, 671500ATB, 671400ATB, 671300ATB, 671200ATB,
Losartan + Hydrochlorothiazide	262500ATB, 378900ATB, 486900ATB
Losartan + Amlodipine	502700ATB, 503000ATB, 513900ATB, 637400ATB, 637500ATB, 637600ATB
Losa+rsvt+ Amlodipine	663900ATB, 664000ATB, 664100ATB, 664200ATB, 664300ATB, 664400ATB,
Losa+chlor+ Amlodipine	662800ATB, 662900ATB, 663000ATB
Fimasartan + Hydrochlorothiazide	522000ATB, 526800ATB
Fimasartan + Amlodipine	651900ATB, 652000ATB, 652100ATB, 652700ATB, 651900ATB
Fimasartan + Rosuvastatin	654600ATB, 654700ATB, 654800ATB, 654900ATB, 655000ATB
Candesartan + Hydrochlorothiazide	423700ATB
Candesartan + Amlodipine	652900ATB, 653000ATB, 653100ATB, 652900ATB, 652900ATB
Candesartan + Rosuvastatin	673700ATB, 661800ATB, 661900ATB, 662000ATB, 662100ATB
Irbesartan + Hydrochlorothiazide	385700ATB, 385800ATB

Irbesartan + Atorvastatin	527000ATB, 527100ATB, 524000ATB, 524100ATB
Azilsartan + Chlorthalidone	673500ATB, 673600ATB
Eprosartan + Hydrochlorothiazide	460500ATB
Calcium carbonate	121801ATB, 121901ATB,
Calcium acetate	121701ATB
Calcium citrate	122101ATB
Paricalcitol	430703BIJ, 430702BIJ, 430701BIJ, 430730BIJ, 430731BIJ
Alfacalcidol	104601ACS, 104601ATB, 104602ACS
Calcitriol	121601ACS, 121602BIJ, 121630BIJ
Calcifediol	121401ACS, 121402ACS
Cinacalcet	512301ATB, 512302ATB
Cholecalciferol + Calcium carbonate	302600ATB, 387900ACS, 409100ATB, 473800ATB, 480200ATB, 498200ATB, 498300ATB, 526100ATB, 634000ATB, 521900ATB
Cholecalciferol + Calcium citrate	462700ATB, 462800ATB, 519000ATB, 503500ATB, 504400ATB, 508700ATB, 665600ATB, 670000ATB
Cholecalciferol + Calcium citrate	503100ATB
Ergocalciferol + Calcium gluconate+ Calcium phosphate	473300ACS
Ergocalciferol + Calcium carbonate+ Calcium gluconate + Calcium lactate	303200ATB
Cholecalciferol + Alendronate	481100ATB, 500200ATB
Cholecalciferol + Ibandronate	523900ATB
Cholecalciferol + Risendronate	511200ATB, 518400ATB
Calcitriol + Alendronate	468000ATE
Cholecalciferol + Ralxifene	659200ACH, 659200ATB
Cholecalciferol + Bazedoxifene	674500ATB
Sevelamer	428501ATB, 428502ATB, 517701APD, 517701ATB
Lanthanum	487101ATB, 487102ATB, 487103ATB, 487104ATB
Atorvastatin	111502ATB, 502202ATB, 633900ATB, 472400ATB, 518900ATB, 524100ATB, 527000ATB, 672000ATR, 672100ATR, 111503ATB, 502203ATB, 634800ATB, 472500ATB, 111504ATB, 502204ATB
Fluvastatin	162401ACH, 162402ACH, 162403ATR
Lovastatin	185801ATB

Pitavastatin	470901ATB, 470902ATB, 470903ATB
Pravastatin	216601ATB, 216602ATB, 216603ATB, 216604ATB
Rosuvastatin	454001ATB, 454002ATD, 454002ATB, 454003ATB, 454003ATD, 454005ATB
Simvastatin	227801ATB, 227802ATB, 227803ATB, 227805ATB, 227806ATB
Atorvastatin + Ezetimibe	633800ATB, 633900ATB, 634800ATB
Pitavastatin + Fenofibrate	679300ACH
Rosuvastatin + Ezetimibe	640700ATB, 640800ATB, 640900ATB
Amlodipine + Atorvastatin	614500ATB, 472300ATB, 472400ATB, 472500ATB, 518900ATB
Amlodipine + Rosuvastatin	673900ATB, 674000ATB, 674100ATB
Aspirin	110701ATB, 110702ATB, 110801ATB, 110802ATB, 111001ACE, 111001ATB, 111001ATE, 111002ATE, 111003ACE, 111003ATE
Aspirin + Bethocarbamol	256800ATB
Aspirin + Clopidogrel	517900ACH, 517900ACE, 517900ATE, 667500ACE
Aspirin + Dipyridamole	489700ACR
Clopidogrel	133201ACR, 133201ATB, 133201ATR, 133202ATB, 133203ATR, 506100ATB

Table S2. Cox regression analyses for cardiovascular event or fracture in subgroups

	Univariable		Multivariable		Univariable		Multivariable		*P* for interaction	
	HR (95% CI)	*P*	HR (95% CI)	*P*	HR (95% CI)	*P*	HR (95% CI)	*P*	Univariable	Multivariable
**Age subgroups**	**Age < 65 years**				**Age ≥65 years**				0.133	0.135
**CVE**										
SH	0.97 (0.81–1.16)	0.715	1.01 (0.83–1.23)	0.915	0.93 (0.73–1.20)	0.580	0.93 (0.71–1.22)	0.587		
ModH	0.91 (0.82–1.01)	0.075	0.93 (0.83–1.04)	0.189	1.01 (0.89–1.14)	0.913	0.93 (0.81–1.06)	0.282		
MilH	0.99 (0.92–1.06)	0.692	0.94 (0.87–1.02)	0.114	0.86 (0.80–0.94)	<0.001	0.86 (0.78–0.94)	<0.001		
UNCa	1.09 (1.03–1.16)	0.002	1.10 (1.04–1.18)	0.003	1.05 (0.98–1.12)	0.155	1.08 (1.00–1.16)	0.048		
HC	1.20 (1.06–1.35)	0.004	1.31 (1.15–1.50)	<0.001	1.20 (1.04–1.40)	0.015	1.25 (1.06–1.48)	0.008		
**Fracture**									0.051	<0.001
SH	0.98 (0.82–1.16)	0.783	1.01 (0.83–1.22)	0.950	0.93 (0.75–1.14)	0.484	1.05 (0.84–1.32)	0.646		
ModH	0.95 (0.86–1.05)	0.343	0.98 (0.88–1.10)	0.773	0.88 (0.79–0.98)	0.015	0.94 (0.84–1.06)	0.316		
MilH	1.01 (0.94–1.09)	0.723	1.01 (0.94–1.09)	0.768	0.88 (0.82–0.94)	<0.001	0.93 (0.86–0.99)	0.049		
UNCa	1.09 (1.03–1.15)	0.002	1.04 (0.98–1.11)	0.216	1.05 (0.99–1.11)	0.101	1.01 (0.95–1.07)	0.808		
HC	1.16 (1.02–1.31)	0.020	1.07 (0.93–1.23)	0.357	0.98 (0.85–1.13)	0.746	0.94 (0.80–1.10)	0.417		
**Sex**	**Male**				**Female**					
**CVE**									0.909	0.631
SH	0.90 (0.76–1.06)	0.198	0.98 (0.82–1.18)	0.846	0.83 (0.62–1.11)	0.201	1.01 (0.74–1.39)	0.927		
ModH	0.91 (0.83–0.99)	0.048	0.93 (0.84–1.03)	0.144	0.87 (0.75–1.02)	0.089	0.96 (0.81–1.14)	0.632		
MilH	0.93 (0.87–0.99)	0.020	0.92 (0.86–0.99)	0.022	0.90 (0.81–0.99)	0.030	0.84 (0.76–0.94)	0.002		
UNCa	1.07 (1.01–1.14)	0.016	1.14 (1.06–1.21)	<0.001	1.03 (0.96–1.10)	0.402	1.06 (0.98–1.14)	0.127		
HC	1.16 (1.01–1.32)	0.037	1.32 (1.13–1.53)	<0.001	1.19 (1.04–1.35)	0.012	1.27 (1.10–1.47)	0.001		

**Fracture**									0.258	0.555
SH	0.98 (0.84–1.15)	0.818	1.07 (0.90–1.28)	0.429	0.78 (0.61–1.00)	0.052	0.92 (0.70–1.21)	0.543		
ModH	0.93 (0.85–1.01)	0.092	0.96 (0.87–1.06)	0.459	0.92 (0.81–1.05)	0.206	0.97 (0.84–1.11)	0.620		
MilH	0.96 (0.90–1.02)	0.173	0.96 (0.89–1.02)	0.187	0.99 (0.92–1.07)	0.864	0.98 (0.90–1.07)	0.665		
UNCa	0.95 (0.90–1.01)	0.098	0.97 (0.91–1.04)	0.394	1.02 (0.96–1.07)	0.537	1.06 (1.00–1.13)	0.039		
HC	1.02 (0.89–1.18)	0.779	1.02 (0.87–1.20)	0.823	0.95 (0.84–1.07)	0.403	1.01 (0.89–1.16)	0.851		
**Diabetes**		**Non–diabetes**				**Diabetes**				
**CVE**									0.337	0.094
SH	0.84 (0.67–1.04)	0.104	0.97 (0.77–1.23)	0.797	0.96 (0.79–1.17)	0.683	1.02 (0.82–1.25)	0.889		
ModH	0.92 (0.82–1.03)	0.161	0.96 (0.84–1.09)	0.507	0.90 (0.81–1.01)	0.067	0.92 (0.81–1.03)	0.155		
MilH	0.89 (0.81–0.96)	0.004	0.88 (0.80–0.96)	0.004	0.92 (0.86–0.99)	0.026	0.92 (0.85–0.99)	0.032		
UNCa	1.13 (1.06–1.19)	<0.001	1.14 (1.06–1.22)	<0.001	1.05 (0.98–1.12)	0.136	1.05 (0.97–1.12)	0.224		
HC	1.24 (1.09–1.39)	<0.001	1.27 (1.11–1.46)	<0.001	1.36 (1.17–1.58)	<0.001	1.35 (1.14–1.59)	<0.001		
**Fracture**									0.715	<0.001
SH	0.85 (0.71–1.03)	0.092	1.04 (0.84–1.28)	0.721	0.90 (0.74–1.09)	0.274	1.05 (0.85–1.28)	0.667		
ModH	0.84 (0.75–0.93)	<0.001	0.91 (0.81–1.02)	0.115	0.93 (0.84–1.02)	0.136	1.03 (0.92–1.15)	0.626		
MilH	0.92 (0.85–0.98)	0.016	0.98 (0.91–1.06)	0.587	0.93 (0.87–0.99)	0.042	0.96 (0.89–1.03)	0.261		
UNCa	1.05 (0.99–1.10)	0.101	1.04 (0.98–1.10)	0.224	1.04 (0.98–1.10)	0.193	0.99 (0.93–1.06)	0.829		
HC	1.08 (0.97–1.21)	0.159	1.02 (0.90–1.16)	0.715	1.01 (0.86–1.19)	0.922	0.95 (0.79–1.14)	0.583		
**Use of VitD**		**No use**				**Use**				
**CVE**									0.274	0.117
SH	0.86 (0.71–1.04)	0.120	0.96 (0.78–1.18)	0.691	0.93 (0.75–1.16)	0.517	1.03 (0.82–1.31)	0.782		
ModH	0.90 (0.81–0.99)	0.049	0.90 (0.80–1.01)	0.080	0.93 (0.82–1.05)	0.215	0.98 (0.86–1.12)	0.791		

MilH	0.87 (0.81–0.94)	<0.001	0.86 (0.80–0.93)	<0.001	0.99 (0.92–1.08)	0.880	0.95 (0.87–1.04)	0.276		
UNCa	1.03 (0.97–1.10)	0.283	1.09 (1.02–1.17)	0.009	1.07 (1.01–1.14)	0.026	1.09 (1.02–1.17)	0.014		
HC	1.12 (0.96–1.30)	0.146	1.27 (1.08–1.50)	0.004	1.25 (1.11–1.42)	<0.001	1.30 (1.13–1.49)	<0.001		
**Fracture**									0.022	<0.001
SH	0.86 (0.72–1.02)	0.089	1.00 (0.82–1.21)	0.969	0.88 (0.72–1.08)	0.211	1.09 (0.88–1.37)	0.428		
ModH	0.93 (0.85–1.03)	0.154	1.03 (0.93–1.14)	0.517	0.80 (0.71–0.90)	<0.001	0.88 (0.77–0.99)	0.042		
MilH	0.94 (0.88–0.99)	0.038	0.98 (0.91–1.05)	0.557	0.93 (0.86–0.99)	0.043	0.95 (0.88–1.03)	0.245		
UNCa	0.98 (0.93–1.04)	0.586	0.98 (0.92–1.04)	0.557	1.08 (1.02–1.15)	0.005	1.06 (0.99–1.13)	0.078		
HC	0.98 (0.85–1.14)	0.789	0.94 (0.80–1.11)	0.447	1.09 (0.97–1.23)	0.147	1.04 (0.91–1.19)	0.539		
**PPB**	**Calcium based PPB**				**Non−user or user of non−calcium based PPB**					
**CVE**									0.479	0.041
SH	0.89 (0.75–1.06)	0.189	1.03 (0.86–1.24)	0.758	0.89 (0.68–1.17)	0.407	0.91 (0.68–1.23)	0.553		
ModH	0.93 (0.85–1.03)	0.157	0.96 (0.86–1.06)	0.383	0.87 (0.75–1.01)	0.071	0.89 (0.76–1.05)	0.169		
MilH	0.94 (0.88–1.00)	0.050	0.93 (0.87–0.99)	0.039	0.90 (0.81–0.99)	0.045	0.83 (0.74–0.93)	0.002		
UNCa	1.02 (0.97–1.08)	0.424	1.03 (0.98–1.10)	0.262	1.08 (1.00–1.17)	0.041	1.23 (1.12–1.34)	<0.001		
HC	1.20 (1.06–1.35)	0.003	1.24 (1.09–1.42)	0.001	1.11 (0.95–1.29)	0.195	1.38 (1.17–1.65)	<0.001		
**Fracture**									0.161	<0.001
SH	0.89 (0.76–1.04)	0.143	1.11 (0.94–1.32)	0.231	0.83 (0.65–1.06)	0.133	0.90 (0.68–1.18)	0.436		
ModH	0.90 (0.83–0.98)	0.018	1.00 (0.91–1.10)	0.974	0.84 (0.74–0.95)	0.008	0.91 (0.79–1.06)	0.229		
MilH	0.92 (0.87–0.98)	0.005	0.98 (0.92–1.04)	0.532	0.97 (0.89–1.05)	0.431	0.95 (0.86–1.04)	0.279		
UNCa	1.06 (1.01–1.11)	0.024	1.01 (0.96–1.07)	0.675	0.96 (0.90–1.03)	0.268	1.02 (0.94–1.11)	0.625		
HC	1.03 (0.92–1.16)	0.612	0.97 (0.85–1.11)	0.672	1.00 (0.86–1.16)	0.999	1.03 (0.87–1.22)	0.714		
**Cinacalcet**	**User**				**None–user**					

**CVE**									0.038	0.001
SH	1.36 (0.64–2.89)	0.422	1.29 (0.53–3.16)	0.579	0.88 (0.76–1.02)	0.078	0.98 (0.83–1.15)	0.790		
ModH	0.74 (0.42–1.30)	0.295	1.06 (0.60–1.87)	0.831	0.91 (0.84–0.99)	0.030	0.93 (0.85–1.01)	0.099		
MilH	0.74 (0.52–1.06)	0.102	0.80 (0.53–1.20)	0.276	0.93 (0.88–0.98)	0.009	0.90 (0.85–0.96)	<0.001		
UNCa	1.30 (1.09–1.56)	0.005	1.15 (0.94–1.42)	0.176	1.05 (1.00–1.10)	0.038	1.09 (1.04–1.15)	<0.001		
HC	1.16 (0.85–1.59)	0.341	1.15 (0.81–1.62)	0.440	1.22 (1.10–1.35)	<0.001	1.33 (1.19–1.48)	<0.001		
**Fracture**									<0.001	<0.001
SH	0.74 (0.30–1.78)	0.495	0.99 (0.41–2.41)	0.986	0.87 (0.76–0.99)	0.044	1.04 (0.90–1.21)	0.603		
ModH	0.24 (0.11–0.54)	<0.001	0.27 (0.11–0.67)	0.004	0.90 (0.83–0.96)	0.003	0.99 (0.91–1.07)	0.720		
MilH	1.04 (0.79–1.36)	0.798	0.95 (0.70–1.30)	0.761	0.93 (0.88–0.98)	0.003	0.97 (0.92–1.02)	0.211		
UNCa	1.17 (0.99–1.37)	0.060	0.98 (0.82–1.17)	0.829	1.03 (0.99–1.07)	0.154	1.02 (0.98–1.07)	0.293		
HC	1.34 (1.04–1.73)	0.026	1.06 (0.79–1.41)	0.714	1.01 (0.92–1.12)	0.792	0.99 (0.89–1.11)	0.897		

Multivariable analysis was adjusted for age, sex, body mass index, diabetes, vascular access, hemodialysis vintage, Charlson comorbidity index score, ultrafiltration volume, Kt/V_{urea}, hemoglobin, serum albumin, serum creatinine, serum phosphorus, use of renin-angiotensin system blockers, cinacalcet, VitD, calcium supplement, aspirin, clopidogrel, PPB, and statins, myocardial infraction or congestive heart failure, and was performed using enter mode. Reference group was Lower normal group.

Abbreviations: CI, confidence interval; CVE, cardiovascular event; HR, hazard ratio; SH, severe hypocalcemia; ModH, moderate hypocalcemia; MilH, mild hypocalcemia; UNCa, upper-normal calcium; UNCa, upper-normal calcium; HC, hypercalcemia; PPB, phosphate binder; VitD, vitamin D analogs.

Table S3. Cox regression analyses for all-cause mortality, cardiovascular event, or fracture based on the quartiles of calcium level.

	Univariable		Multivariable	
	**HR (95% CI)**	***P***	**HR (95% CI)**	***P***
**All-cause mortality**				
2Q	1.09 (1.06–1.13)	<0.001	1.04 (1.00–1.07)	0.044
3Q	1.09 (1.05–1.12)	<0.001	1.11 (1.07–1.15)	<0.001
4Q	1.09 (1.06–1.13)	<0.001	1.21 (1.17–1.26)	<0.001
**Cardiovascular event**				
2Q	1.07 (1.01–1.12)	0.013	1.06 (1.00–1.12)	0.047
3Q	1.06 (1.01–1.11)	0.025	1.10 (1.04–1.16)	<0.001
4Q	1.13 (1.08–1.19)	<0.001	1.22 (1.15–1.29)	<0.001
**Fracture**				
2Q	1.06 (1.01–1.11)	0.010	1.01 (0.96–1.06)	0.651
3Q	1.12 (1.07–1.17)	<0.001	1.06 (1.01–1.11)	0.020
4Q	1.12 (1.07–1.17)	<0.001	1.06 (1.01–1.12)	0.017

Multivariable analysis was adjusted for age, sex, body mass index, diabetes, vascular access, hemodialysis vintage, Charlson comorbidity index score, ultrafiltration volume, Kt/V_{urea}, hemoglobin, serum albumin, serum creatinine, serum phosphorus, use of renin-angiotensin system blockers, cinacalcet, vitamin D analogs, calcium supplement, aspirin, clopidogrel, phosphate binder, and statins, myocardial infraction or congestive heart failure. It was performed using enter mode. The 1Q group served as the reference.

Abbreviations: CI, confidence interval; HR, hazard ratio; 1Q, first quartile; 2Q, second quartile; 3Q, third quartile; 4Q, fourth quartile.

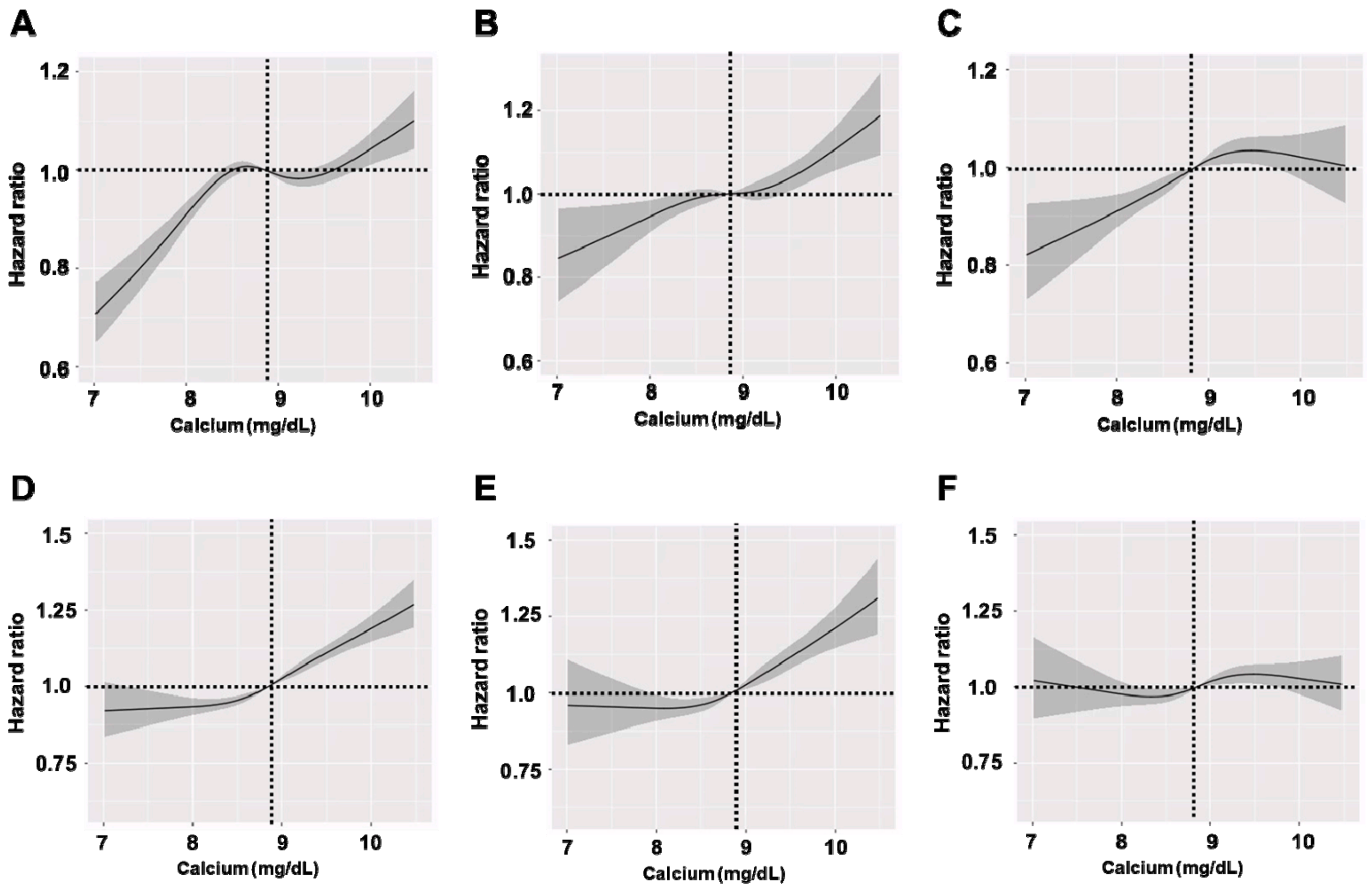

Figure S1. Spline curves show the hazard ratios and 95% confidence intervals of clinical outcomes according to calcium level (A and D, all-cause mortality; B and E, cardiovascular events; C and F, fracture). Panels A–C show univariable analyses; panels D–F show multivariable analyses. The reference point was established at a median calcium level of 8.85 mg/dL. Data were plotted using a uni- or multivariable model. Adjustments were made in terms of the following factors: age, sex, body mass index, diabetes, vascular access, hemodialysis vintage, Charlson comorbidity index score, ultrafiltration volume, Kt/V_{urea}, hemoglobin, serum albumin, serum creatinine, serum phosphorus, use of renin-angiotensin system blockers, cinacalcet, vitamin D analogs, calcium supplement, aspirin, clopidogrel, phosphate binder, and statins, myocardial infraction or congestive heart failure.

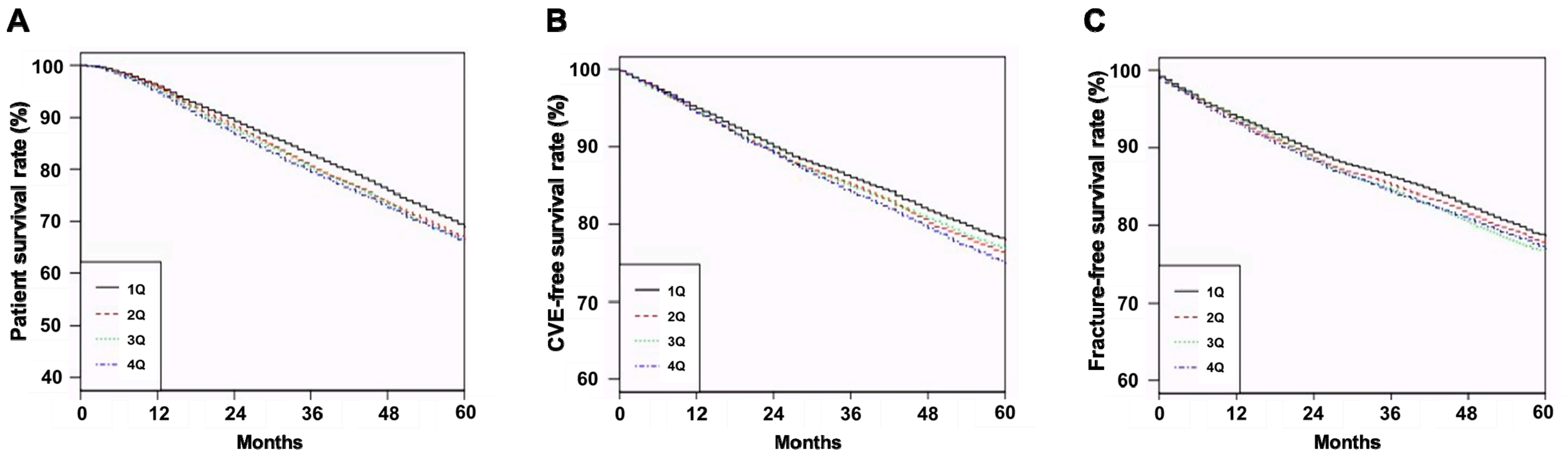

A – Number at risk

	0	12	24	36	48	60
1Q	17,526	16,706	15,383	14,070	9,014	8,063
2Q	17,525	16,690	15,186	13,738	9,103	8,136
3Q	17,525	16,573	15,045	13,597	9,352	8,329
4Q	17,525	16,490	14,883	13,414	10,118	9,031

	1Q	2Q	3Q
2Q	<0.001		
3Q	<0.001	0.830	
4Q	<0.001	0.810	0.640

B – Number at risk

	0	12	24	36	48	60
1Q	12,606	11,588	10,395	9,312	5,942	5,250
2Q	12,575	11,502	10,179	9,083	5,986	5,269
3Q	12,763	11,598	10,274	9,118	6,325	5,568
4Q	13,158	11,929	10,519	9,284	6,970	6,119

	1Q	2Q	3Q
2Q	0.025		
3Q	0.032	0.791	
4Q	<0.001	0.025	0.023

C – Number at risk

	0	12	24	36	48	60
1Q	17,526	15,785	13,944	12,428	7,638	6,622
2Q	17,525	15,681	13,713	12,049	7,673	6,626
3Q	17,525	15,609	13,547	11,827	7,817	6,751
4Q	17,525	15,450	13,362	11,642	8,524	7,371

	1Q	2Q	3Q
2Q	0.021		
3Q	<0.001	0.023	
4Q	<0.001	0.031	0.949

Figure S2. Kaplan–Meier curves of patient survival, cardiovascular events, or fracture according to the quartiles of calcium level. (A) Patient survival. (B) Cardiovascular events. (C) Fracture. *P*-values for pairwise comparison with log-rank tests were added to the bottom of the graph.

Abbreviations: CVE, cardiovascular events; 1Q, first quartile; 2Q, second quartile; 3Q, third quartile; 4Q, fourth quartile.
